# Aluminum Alloys

**Published:** 1894-03

**Authors:** N. K. Garhart

**Affiliations:** Indianapolis.


ARTICLE IX.

### ALUMINUM ALLOYS.

BY N. K. GARHART, INDIANAPOLIS.

Of all the glittering absurdities and alluring fakes known to dentistry, the introduction of aluminum alloys maintains supremacy in the art of swindling. My attention has been frequently called to the extensive advertisements of aluminum alloy manufacturers by a number of dentists who were anxious to learn if their statements were valid. As a sample of glaring imposition, a Chicago firm claims that its alloy contains 60 per cent. aluminum, a manifest absurdity in an alloy designed for dental use. In spite of the excellent sources of information concerning the chemistry of metals which our dental colleges afford, many dental practitioners insist on believing the wild and erroneous statements of these manufacturers.

The following rule will aid those who are insufficiently informed on the essential properties of a practical alloy for dental use:

All metals which do not readily unite with mercury are detrimental to alloys; if such metals are found or advertised to be constituents of alloys, avoid purchasing.

Amalgamation of aluminum can be effected indirectly by several methods. An amalgam thus formed is extremely unstable. On exposure to air the amalgam suffers change, the aluminum rapidly oxidizes enveloping the surface of the separated mercury in the form of a white oxid. The production of heat is always concomitant with the chemical union of any metal with oxigen. The amalgam when thrown into water decomposes it with the evolution of hydrogen and precipitation of mercury and hydrated oxid of aluminum. This reaction is similar to the action of metallic sodium on water.

The methods for amalgamating aluminum are as follows:

(1.) Triturating fragments of the metal with mercury in the presence of solutions of the hydrates of sodium and potassium.

(2.) Electrolytically by connecting a strip of aluminum with the negative pole of a battery and immersed in mercury.

(3.) By pouring together melted aluminum and mercury previously brought to the boiling point.

The following experiment illustrates the value of aluminum for a dental alloy:

Forty parts of silver, fifty tin, and ten aluminum were compounded to form an alloy; during the formation of the alloy a portion of the aluminum separated, the amount of which was not ascertained. The alloy was reduced to filings, and a portion triturated with mercury in a glass mortar; as amalgamation progressed decomposition set in, the mercury separating in the form of a globule from a large quantity of black powder. The experiment was repeated, using the palm of the hand and forefingers as implements to facilitate the mixing. On completion of the mixing the mass grew suddenly hot, and in less than a second I was forced to drop it. The experiment was repeated a third time with the same results.

The next step was to adduce a theory to explain the above phenomena; also why mercury will not unite directly with aluminum.

Aluminum possesses a marked affinity for oxigen. This affinity is so great that the metal under proper condition is capable of decomposing water in precisely the same manner as the metals lithium, sodium, potassium and calcium. This statement may appear absurd when we observe that aluminum is rather permanent in air and water. However, that fact does not overthrow the theory. Aluminum is protected from the action of air and moisture by a thin oxid of the metal covering its entire surface. The metal and its oxid surface is soluble in solutions of the alkalies, and hydrochloric and sulfuric acids. This oxid being insoluble in water protects the aluminum from oxidizing in the presence of moisture and air. It also explains why the metal will not unite directly with mercury. This film of oxid protects the metal from direct contact with the mercury. The oxids of sodium, lithium and calcium are very soluble in water, hence the speedy decomposition of these metals in water are easily accounted for. If these metals are covered with a thin film of a hydrocarbon, such as coal oil or vaselin, they are then unaffected by air. Brass ornaments are protected from the corrosive action of air and moisture by coating the burnished metal with transparent varnish.

On triturating fragments of aluminum with mercury, combination of the metals does not occur, but if a solution of an alkali is added amalgamation of the aluminum is effected. The alkali dissolves the film of oxid of aluminum, and the two metals are constantly in contact with each other. By melting the aluminum the oxid rises to the surface, protecting it from further oxidation. On adding the boiling mercury it sinks to the bottom of the vessel underlying the melted metal, owing to its high specific gravity. The metals which are in contact with each other unite to form an amalgam.

The affinity existing between aluminum and mercury is very feeble. The aluminum spontaneously separates from the mercury in an exceedingly fine state of division. So minute are the particles of the metal that every portion is exposed to the energetic action of the oxigen. The influence of oxigen induces the separation of aluminum.

The phenomena attending the "alloy experiment" are rather interesting. The aluminum is oxidized, and the silver, tin, and possibly small quantities of mercury are also converted into oxids. The absorption of oxigen producing oxidation of the aluminum, induces oxidation of the metals, silver and tin, which have a feeble affinity for that element.

The latter part of the reaction is partly induced by the elevation of temperature resulting from the combustion of aluminum in oxigen. Finely divided aluminum resembles the metal platinum, which possesses the peculiar property of absorbing oxigen with avidity when existing in a fine state of division, and is incapable of undergoing oxidation owing to the feeble attraction of one element for the other. A stream of hydrogen directed on finely divided platinum (platinum black) will clearly demonstrate that wonderful property The occluded oxigen energetically attacks the hydrogen which ignites, and the harmless product water is formed. It is possible that many metals, when existing in a fine state of division, may possess the same properties. Is it not possible for aluminum, when existing in a fine state of division, to absorb oxigen in a similar manner, undergoing oxidation, and at the same time oxidizing the metals silver and tin? It appears reasonable that aluminum possesses "platinum black" properties, or the influence of the metal produces a peculiar molecular arrangement including oxidation of the other metals.—*Items of Interest.*

